# Flattening the curve is flattening the complexity of covid-19

**DOI:** 10.1007/s40656-021-00374-x

**Published:** 2021-02-10

**Authors:** Marcel Boumans

**Affiliations:** Utrecht University, Utrecht, Netherlands

**Keywords:** Compartmental model, Epidemic control, Reproduction number, Shape of an epidemic curve, Social distancing

## Abstract

Since the February 2020 publication of the article ‘Flattening the curve’ in *The Economist*, political leaders worldwide have used this expression to legitimize the introduction of social distancing measures in fighting Covid-19. In fact, this expression represents a complex combination of three components: the shape of the epidemic curve, the social distancing measures and the reproduction number $\mathscr{R}_{0}$. Each component has its own history, each with a different history of control. Presenting the control of the epidemic as flattening the curve is in fact flattening the underlying natural-social complexity. The curve that needs to be flattened is presented as a bell-shaped curve, implicitly suggesting that the pathogen’s spread is subject only to natural laws. The $\mathscr{R}$ value, however, is, fundamentally, a metric of how a pathogen behaves within a social context, namely its numerical value is affected by sociopolitical influences. The jagged and erratic empirical curve of Covid-19 illustrates this. Although the virus has most likely infected only a small portion of the total susceptible population, it is clear its shape has changed drastically. This changing shape is largely due to sociopolitical factors. These include shifting formal laws and policies, shifting individual behaviors as well as shifting various other social norms and practices. This makes the course of Covid-19 curve both erratic and unpredictable.

## Introduction

**Fig. 1:**
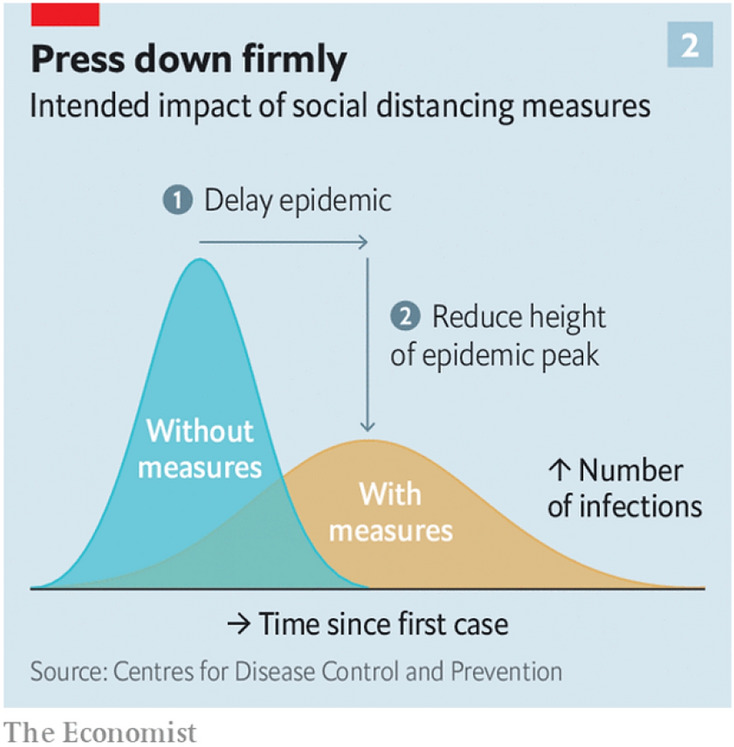
Chart 2 of the article in *The Economist* showing how the course of an epidemic is shaped by the reproductive rate. This chart was adapted from a figure published in CDC 2007 (see Figure 3). *Source*: Economist 2020

In an early stage of the Covid-19 pandemic, in February 2020, *The Economist* (Vol. 434, Issue 9183) published the article ‘Flattening the curve’. This article discussed the economic consequences of social distancing. The impact of the social distance measures on the assumed development of the epidemic was illustrated in a chart (see Fig. 1).

Although the chart displays a direct link between the social distancing measures and the shape of the curve, in the corresponding text the impact was presented more indirectly, namely through a variable labeled $\mathscr{R}$: “the course of an epidemic is shaped by a variable called the reproductive rate, or $\mathscr{R}$.” As a result, the *Economist* article connected three different components: the shape of the epidemic curve, the social distancing measures and the “reproductive rate $\mathscr{R}$.” Actually, it is the combination of all three components that is captured by the slogan “flatten the curve”: “To flatten the curve you must slow the spread.”^1^

Since the publication of the article, ‘to flatten the curve’ has become the common expression used by political leaders worldwide in legitimizing the introduction of social distancing measures in fighting Covid-19. This policy is presented in a visually strong and clear way, and is therefore easy to comprehend: the epidemic is visualized through a bell-shaped curve that needs to be flattened. It is the contrast between this complex societal problem with its many economic, social, political, psychological, and medical dimensions and the simple visual representation of it which made me wonder where it came from. This article is the result of my research.

The concept of ‘flatten the curve’ is a nice example of the notion of controlling a macro-phenomenon. This notion is based on two implicit assumptions: the epidemic phenomenon apparently has a specific shape and this shape appears to have such materiality that it can be shaped. As a result, control of a macro-phenomenon means re-shaping the shape of the phenomenon left uncontrolled.^2^ This article analyses this kind of control by exploring how it arose and how it received this specific meaning. To do this, the origins of each component have to be traced back. It appears that each component has its own history, each with a different history of control. It is only when they were integrated that control acquired its current meaning of shaping a curve.

## The curve of a happening

**Fig. 2:**
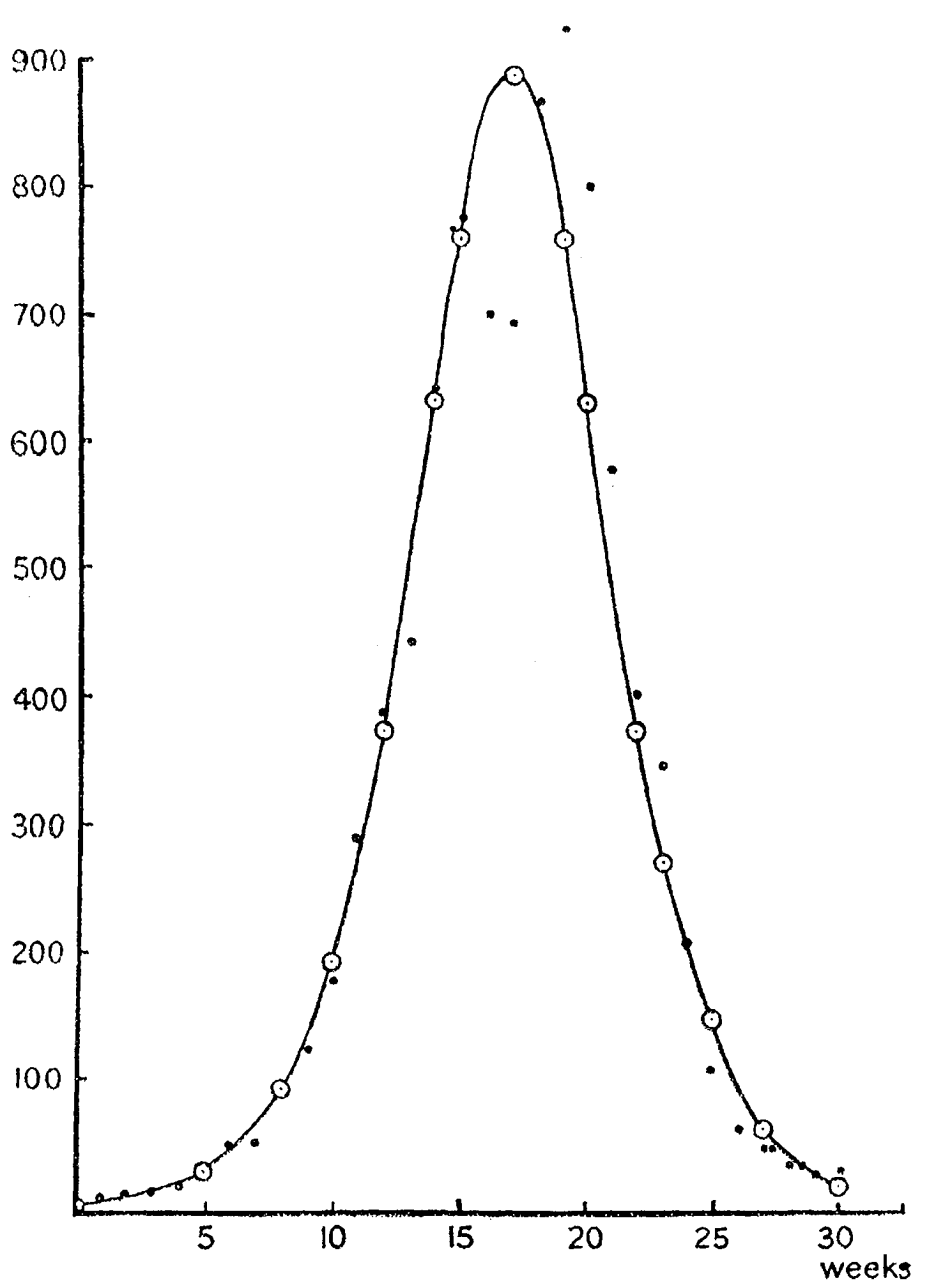
Deaths from plague in the island of Bombay over the period December 17, 1905, to July 21, 1906. The ordinate represents the number of death per week, and the abscissa denotes the time in weeks. The “calculated curve” is drawn from the formula $\frac{dR}{{dt}} = 890{\text{sech}}^{2} \left( {0.2t - 3.4} \right)$. (Source: Kermack and McKendrick 1927, p. 714)

The curve of an epidemic was presented for the first time (see Fig. 2) in the article ‘A contribution to the mathematical theory of epidemics,’ published in 1927 and written by William Ogilvy Kermack and Anderson Gray McKendrick. This work was inspired by Ronald Ross. Ross’s ideas about applying mathematical reasoning to infectious disease dynamics originated from his ambition to understand malaria transmission and control. He was the first to develop a general theory of epidemic phenomena (which he called a ‘theory of happenings’) of infectious disease dynamics. This theory was not specifically tailored to a particular pathogen or public health problem but based on prior assumptions about mechanisms that could be acting in the spread of infections (rather than trying to obtain insight a posteriori by studying real epidemics) (Heesterbeek and Roberts 2015, p. 2; Heesterbeek 2002, pp. 192–3).

This first figure of an epidemic curve was the result of a new development in epidemiology at the beginning of the twentieth century, namely the development of mathematical compartmental models. In these models, the population under study is divided into compartments. Assumptions are made about the nature and time rate of transfer from one compartment to another. For example, in the currently well-known *SIR* model the population is divided into three classes labelled *S*, *I*, and *R*. Here *S*(*t*) denotes the number of individuals who are susceptible to the disease, that is, who are not (yet) infected at time, *t*; *I*(*t*) denotes the number of infected individuals, assumed infectious and able to spread the disease by contact with susceptibles; and *R*(*t*) denotes the number of individuals who have been infected and then removed from the possibility of being infected again or of spreading infection.

The first epidemic models to describe the transmission of communicable diseases were developed in a sequence of three papers by Kermack and McKendrick, of which the first (1927) provided a compartmental model (Brauer 2017, p. 114). The situation they modelled was described as follows:

One (or more) infected person is introduced into a community of individuals, more or less susceptible to the disease in question. The disease spreads from the affected to the unaffected by contact infection. Each infected person runs through the course of the sickness, and finally is removed from the number of those who are sick, by recovery or by death. […] As the epidemics spreads, the number of unaffected members of the community becomes reduced. Since the course of an epidemic is short compared with the life of an individual, the population may be considered as remaining constant, except in so far as it is modified by deaths due to the epidemics itself. In the course of time the epidemic may come to an end. […] In the present communication discussion will be limited to the case in which all members of the community are initially equally susceptible to the disease, and it will be further assumed that complete immunity is conferred by a single infection. (Kermack and McKendrick 1927, pp. 700–701).

The resulting model was a *SIR* model, though expressed in the variables *x*, *y*, and *z*.^3^ As the initial population density, *N*, was assumed to be constant, the following relation applies: *S*(*t*) + *I*(*t*) + *R*(*t*) = *N*. Kermack and McKendrick also defined two relevant rates: *γ*_*θ*_, the rate of removal, that is the sum of the recovery and death rates at age *θ*; and *β*_*θ*_ is the rate of infectivity at age *θ*.

This general case led to integral equations. These are difficult or even impossible to be solved analytically, in the sense that a function can be given as exact solution. These equations are usually solved by numerical methods. However, Kermack and McKendrick’s “calculated curve” (Fig. 2) is an analytical solution based on the simplifying assumptions that *β*_*θ*_ and *γ*_*θ*_ are the constants *β* and *γ* respectively. In this case the dynamics of the epidemic can be described by the following three equations:

1 $$\frac{dS}{{dt}} = - \beta SI$$

2 $$\frac{dI}{{dt}} = \beta SI - \gamma I$$

3 $$\frac{dR}{{dt}} = \gamma I$$

The solution of these three equations can be expressed in terms of the rate at which cases are removed by death or recovery (*dR/dt*) “which is the form in which many statistics are given” (p. 714). Even though these equations still cannot be solved analytically, McKendrick and Kermack provided a function as an approximation to the solution:

$$\frac{dR}{{dt}} = \frac{{\gamma^{3} }}{{S_{0} \beta^{2} }}q{\text{sech}}^{2} \left( {q\gamma t - \beta } \right),$$

where *q* is a function of *β*, *γ*, *S*_0_, and *I*_0_. In other words the shape of an epidemic was presented as the shape of the sech^2^ curve (see Fig. 2).^4^

Although the Eqs. (1)–(3) do not have a function as analytic solution, graphical representations of numerical solutions also show bell-shaped curves, however, these are not symmetrical. Nonetheless, it is Kermack and McKendrick’s symmetrical shape that came to be commonly used to represent the curve of an epidemic.

The possibility of control, if any, was conceived in terms of critical community size. Ross proved that not all mosquitos needed to be eliminated to stop the malaria parasite from spreading, but that the depression of the number of mosquitos per human host in a population to a value below a critical level was sufficient. McKendrick and Kermack generalized Ross’s initial ideas of critical thresholds for malaria to be a critical size of a community of susceptible individuals necessary for an infectious disease to become established in a population (Heesterbeek and Roberts 2015, p. 2).

## $\mathscr{R}_{0}$

The concept of the $\mathscr{R}_{0}$ did not originate in epidemiology and has a more complicated cross-disciplinary history than the epidemic curve.

The concept of $\mathscr{R}_{0}$ is closely linked to quantities such as ‘net fertility’ or ‘net reproductive rate’ in demography (introduced mainly through the work of Alfred Lotka), and ‘absolute fitness’ or ‘reproductive fitness’ in population genetics (introduced mainly through the work of Ronald Fisher and Sewall Wright), although these concepts did not evolve from each other in a linear manner. They all describe the average contributions of members of a given generation to the next generation, in terms of new infections caused, the birth of daughters, or genotypes produced. (Heesterbeek and Roberts 2015, p. 2).

An importance source of the history of $\mathscr{R}_{0}$ is Hans Heesterbeek’s (2002) ‘brief history of $\mathscr{R}_{0}$.’ Although $\mathscr{R}_{0}$ is “arguably the most important quantity in the study of epidemics and notably in comparing population dynamical effects of control strategies,” Heesterbeek (2002, p. 189) emphasizes that “the use of $\mathscr{R}_{0}$ in its present form is of relatively recent origin in epidemiology.” Heesterbeek gives the following explanation for $\mathscr{R}_{0}$ achieving this prominent role in modern epidemiology:

It took a long time for modellers in epidemiology to realise that the formulation in terms of reproduction potential is a much clearer and more powerful concept for infectious diseases as well, which is moreover much more amenable to generalisation to heterogeneous populations, and can be tied much more easily to data and hence applications. An important reason for this long delay in epidemiology […] can indeed be this link to data. The early development of $\mathscr{R}_{0}$ in ecology/demography had a much closer link to empiricism than the early development in epidemiology in the hands of Kermack and McKendrick and others, who were much more interested in presenting a mathematically coherent theory. After the realisation, around 1975, that the reproduction potential was to be preferred over critical size, the major hurdle that had to be taken was to tie the formal concept to empiricism. The use of $\mathscr{R}_{0}$ finally took off when it was found that the quantity could be estimated from readily available data. (Heesterbeek 2002, p. 190–1).

According to Heesterbeek, it is mainly due to the work of Roy Anderson and Robert May in the 1970s and 1980s that $\mathscr{R}_{0}$ gained this prominent role. In 1979, they published a two-part paper on the population biology of infectious diseases in *Nature*. While this paper “played a dominant role in revitalising the subject of infectious disease modelling, after attention for it had waned from the late nineteen-sixties” (p. 199), the concept of $\mathscr{R}_{0}$ is not used. The entire analysis is done in terms of critical sizes of host populations. However, a few years later they published a paper in *Science* which made extensive use of $\mathscr{R}_{0}$. In this 1982 paper and at a conference in the same year, they promoted the application of $\mathscr{R}_{0}$ in epidemiology. These contributions were “most influential in reviving scientific interest in applying mathematical modelling as a tool in studying the spread and control of infectious agents” (p. 199).

The aim of the *Science* article was “to show how relatively simple models can provide a broad biological understanding of the factor controlling disease persistence and recurrent epidemic behavior (including the changes wrought by specific vaccination programs), and on how they can make detailed contact with data” (Anderson and May 1982, p. 1054). The basic model explored in this article was a compartmental model consisting of four first-order differential equations describing the dynamics of the infection within its host population, a *SEIR* model:

$$\begin{gathered} dS/dt = \mu N{-}\mu S{-}\beta SI \hfill \\ dE/dt = \beta SI{-} \, (\mu + \sigma)E \hfill \\ dI/dt = \sigma E{-} \, (\mu + \gamma)I \hfill \\ dR/dt = \gamma I{-}\mu R \hfill \\ \end{gathered}$$

*E* is the class of the exposed, the infected who are not yet infectious. *μ* is the birth rate and is assumed to be equal to the death rate. The net rate at which infections are acquired is proportional to the number of encounters between susceptibles and infectious individuals, *βSI*, where *β* is a transmission coefficient. Individuals pass from the latent state to the infectious state at a per capita rate *σ* and recover to join the immune class at a per capita rate *γ*.

Immediately after the presentation of this model, the $\mathscr{R}$ was introduced in the following way:

The disease will maintain itself within the population provided the “reproductive rate,” $\mathscr{R}$, of the infection is greater than or equal to, unity; $\mathscr{R}$ is the expected number of secondary cases produced by an infectious individual in a population of *S* susceptibles. (Anderson and May 1982, p. 1054).

Based on the above model, the formal expression of $\mathscr{R}$ was $\frac{\sigma \beta S}{{\left( {\sigma + \mu } \right)\left( {\gamma + \mu } \right)}}$, which can be interpreted as secondary infections are produced at a rate *βS* throughout the expected lifetime, 1/(*γ* + *μ*), of the infectious individual; of these, a fraction *σ*/(*σ* + *μ*) will survive the latent period to become the second generation of infectious individuals. $\mathscr{R}_{0}$, the ‘intrinsic reproductive rate” was defined as the value of $\mathscr{R}$ in a disease-free population. That is to say, assuming no vaccination and all individuals are susceptible, *S* = *N*.

Anderson and May (1982, p. 1055) emphasized that “the concept of the intrinsic reproduction rate, $\mathscr{R}_{0}$, is central to an understanding both of the epidemiology of infectious diseases and of the impact of control policies.” The control policy was conceived as reducing $\mathscr{R}_{0}$ below unity. The way to achieve this was by immunizing a proportion, *p*, of the population by vaccination soon after birth, such that $p > \, 1 \, {-} \, \left( {1/{\mathscr{R}}_{0} } \right)$.

Although Anderson and May (1982) introduced $\mathscr{R}_{0}$ to epidemiology as a concept to understand how to design control policies, it was only discussed in terms of population densities, even though the formal expression of $\mathscr{R}$ hinted at other options of control. Some of the parameters determining $\mathscr{R}$ were specific to the disease agent. The examples Anderson and May mentioned were *σ* and *γ*, and also *β* in its relationship to “the expected life-span of the infected particle or spore in the external environment” (p. 1054). However, other components of $\mathscr{R}$, such as the density of susceptibles *S*, and the parameter *β*, could also be looked upon as reflecting “the average frequency of contacts between individuals” depended on “the prevailing environmental and social conditions.” They also mentioned “even the value of 1/*γ* may be influenced by such conditions, since the isolation of infected children can substantially reduce the effective infectious period” (p. 1054). However, control in terms of influencing these social conditions were not further discussed in the article.

Previous literature, such as discussed above, uses $\mathscr{R}$ and $\mathscr{R}_{0}$ interchangeably. However, it should be emphasized that in the current epidemic literature both symbols have a fixed meaning. $\mathscr{R}_{0}$ is the “basic reproduction number,” and is defined as the expected number of secondary cases produced by a single (typical) infection in a completely susceptible population. Although the variable was initially referred to as “reproductive rate,” it was later pointed out that it is neither reproductive nor a rate, it is a dimensionless number (see below). Hence the current preferred name in the epidemic literature is “reproduction number.”^5^$\mathscr{R}$ does not depend on the assumption that the population is completely susceptible. This assumption is often violated in the later stages of an outbreak or in a situation in which the population has been previously exposed to the pathogen.

## Social Measures

Despite May and Anderson successfully advocating the use and the value of $\mathscr{R}_{0}$ in the early 1980s, it took a number of years before epidemiologists realized its potential (Heesterbeek 2002, p. 199). The perception of $\mathscr{R}_{0}$ and how it can enlighten adequate policy measures changed only with the SARS epidemic of 2002–3, “despite rapid early spread, the epidemic eventually was contained, reflecting in part a highly effective global public health responses” (Fraser e.a. 2004, p. 6146). In the article ‘Factors that make an infectious disease outbreak controllable’ the methods used to control SARS were assessed as “likely to be equally effective for future outbreaks of other emerging infectious […] even when effective vaccines or treatment are not available” (p. 6146). The article aimed to understand the social factors that make containment feasible.

Two “basic” public health policy options “in the absence of effective vaccines or treatment” were explored: “(*i*) effective isolation of symptomatic individuals and (*ii*) tracing and quarantining of the contacts of symptomatic cases” (Fraser e.a. 2004, p. 6146). As a result of this, three important parameters were identified:

1. The “basic reproduction number” $\mathscr{R}_{0}$.
2. The “disease generation time,” the mean time interval between the infection of one person and the infection of the people that individual infects.
3. The “proportion of transmission occurring prior to symptoms (or asymptotically)” *θ* which determines the potential for symptom-based public health control measures to reduce the number of infections. (p. 6146)

The analysis was based on an “idealized optimal intervention,” that is to say, without delays in implementation of isolation and quarantining. This meant that the disease generation time did not play an important role. Delays if needed, could be taken into account by *θ*. The result of the analysis was “that the interventions are sufficient to control outbreaks of infections for combinations of values of parameters $\mathscr{R}_{0}$ and *θ* falling below a certain critical line” (p. 6147).

As a result of the SARS epidemic and the concern of a possible H5N1 influenza epidemic in 2005, scientists and policymakers were growing more concerned that the world may soon face a pandemic. One in which neither vaccines nor sufficient antivirals would be available to protect the public. For this reason, in the US, a Committee on Modeling Community Containment for Pandemic Influenza was set up to investigate if “nonpharmaceutical community containment strategies may help in the absence of sufficient medical interventions” (Mahmoud 2006, p. 1). Six mathematical models were used to evaluate the role of “nonpharmaceutical interventions” in mitigating a pandemic influenza outbreak. The main focus was the “measure of infectivity,” $\mathscr{R}_{0}$, “the average number of secondary cases of disease generated by a typical primary case in a susceptible population” (p. 2).

These evaluations led to the conclusion that “evidence suggests a role for surveillance and case reporting, rapid viral diagnosis, hand hygiene, and respiratory etiquette in reducing pandemic influenza virus transmission” (p. 24). The results also suggested “a role for contact tracing (early in the epidemic) to allow for individual action by the contact, voluntary sheltering, and quarantine in reducing pandemic influenza virus transmission” (p. 27). The report provided 11 recommendations, of which the 9th reveals the kind of evidence on which the conclusions were based:

The committee recommends that communication regarding possible community interventions for pandemic influenza that flows from the federal government to communities and from community leaders to public not to overstate the level of confidence or certainty in the effectiveness of these measures. The communications should also not overstate the role that modeling or historical analyses play in supporting these interventions. (Mahmoud 2006, pp. 29–30).

## Shaping the epidemic curve

While in earlier publications a connection between $\mathscr{R}$ and the shape of the epidemic curve was already suggested, such as in the Anderson and May 1981 article, “it is immediately evident that the dynamics of the infection, the shape of the [epidemic] curve […], depends only on the quantity $\mathscr{R}$” (p. 460), and in Mahmoud’s (2006) Letter Report the conclusion that the effect of early interventions “might be to slow the time to peak of the outbreak in a community,” the explicit connection was only made in a ‘Interim Pre-pandemic Planning Guidance’ published in 2007.

The Guidance was developed to plan and prepare for “the first wave of the next pandemic without vaccine and potentially without sufficient quantities of influenza antiviral medications” (CDC 2007, p. 8). It formulated the following rationale for Non-Pharmaceutical Interventions (NPIs):

(1) delay the exponential increase in incident cases and shift the epidemic curve to the right in order to “buy time” for production and distribution of a well-matched pandemic strain vaccine, (2) decrease the epidemic peak, and (3) reduce the total number of incident cases and, thus, reduce morbidity and mortality in the community. (CDC 2007, p. 9).

**Fig. 3:**
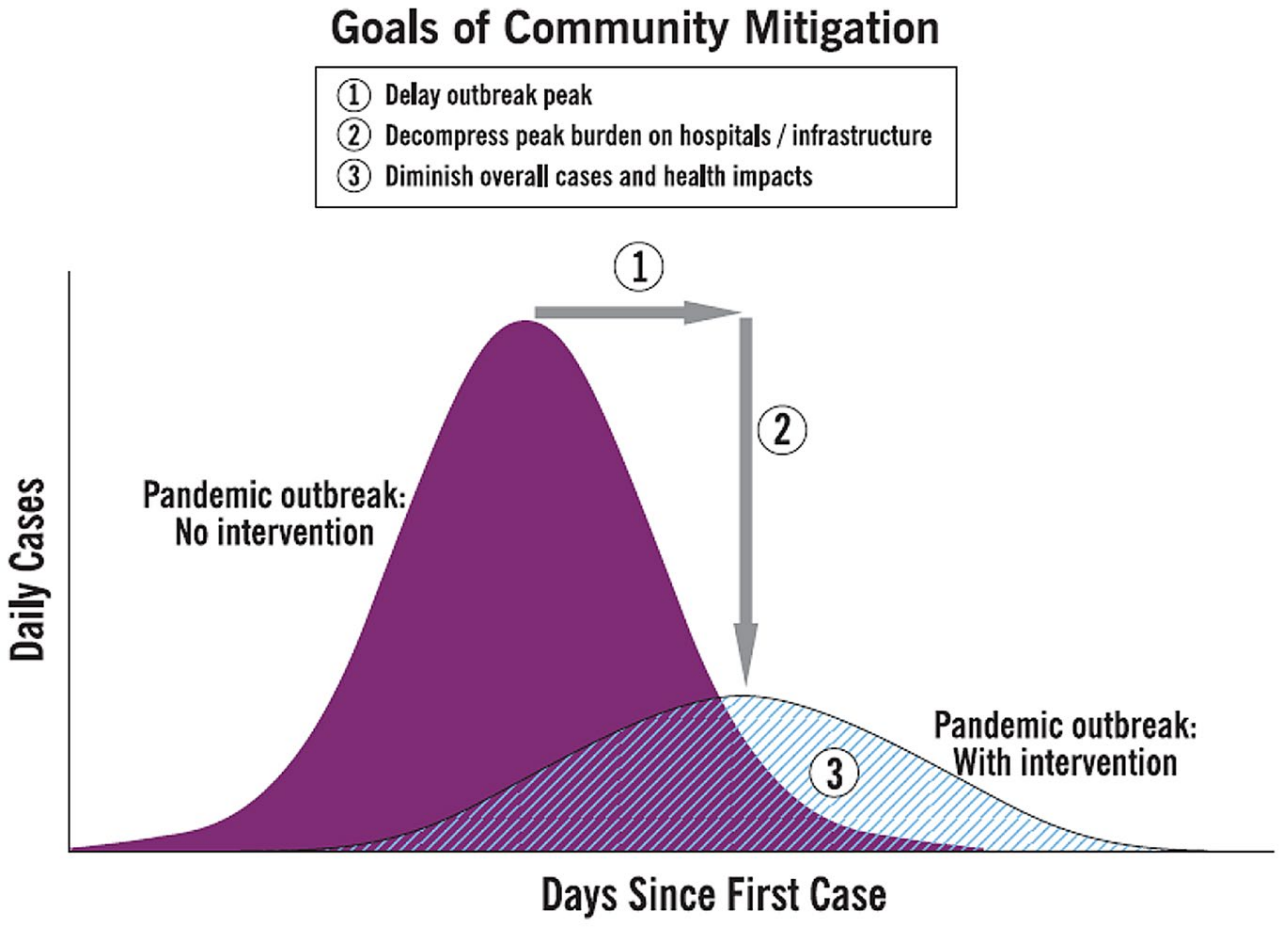
Source: CDC 2007, p. 18

This rationale was illustrated with the following figure (Fig. 3):

This figure is the one that was reproduced in *The Economist* of February 2020, however, without the 1, 2, 3 numbering scheme and with different colors. Rosamund Pearce, a data visualization journalist at *The Economist*, decided to rebuild it for this article on Covid-19. Her words “I thought it was a beautifully clear and simple illustration of an important concept” (Wilson 2020). Pearce kept the graphic as close to the original in terms of shape as she could, because.

"The difficulty with these diagrams is showing uncertainty. Even though it’s a diagram of a concept and not a model from real data, it’s easy for people to interpret it as a precise prediction, as it looks like a chart and we’re used to charts being precise,” says Pearce. “Once you’ve drawn these shapes, they look authoritative, even if they’re intended to be illustrative. That’s why I keep as close to the CDC’s as I could.” (Wilson 2020).

**Fig. 4:**
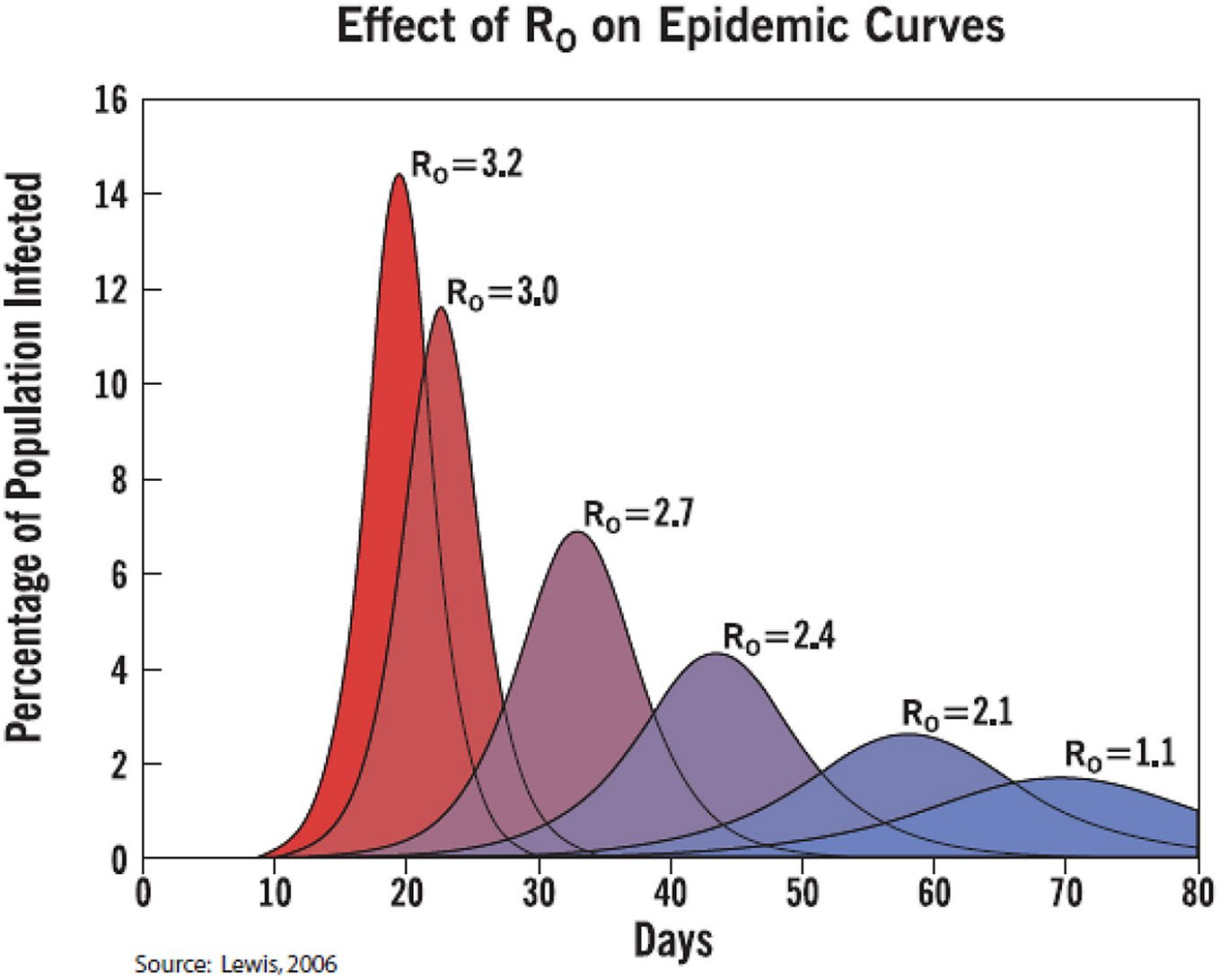
Source CDC 2007, p. 24

The connection with $\mathscr{R}$, however, was most explicitly made in another CDC figure (See Fig. 4). This figure was to illustrate a specific property of $\mathscr{R}_{0}$: “$\mathscr{R}_{0}$ is not an intrinsic property of the infectious agent but rather an epidemic characteristic of the agent within a specific host within a given milieu. […] Alterations in the pathogen, the host, or the contact networks can result in changes in $\mathscr{R}_{0}$ and thus in the shape of the epidemic curve (CDC 2007, p. 23).

According to the *Guidance*, the value of $\mathscr{R}_{0}$ can be influenced by various “pandemic mitigation strategies”. For example:

(1) case containment measures, such as voluntary case isolation, voluntary quarantine of members of households with ill persons, and antiviral treatment/prophylaxis, (2) social distancing measures, such as dismissal of students from classrooms and social distancing of adults in the community and at work, and (3) infection control measures, including hand hygiene and cough etiquette. (CDC 2007, p. 28).

To determine if these measures were successful, mathematical models were used to assess various types of interventions within the context of social networks. These simulations suggested that “a combination of targeted antiviral medications and NPIs can delay and flatten the epidemic peak” (p. 29).

The relationship between the shape of the curve, $\mathscr{R}$, social distancing and the idea of ‘flattening the curve,’ originates in this CDC report. Interestingly, a similar reservation was made regarding the confidence in the effectivity of social measures:

Taken together, these strands of evidence are consistent with the hypothesis that there may be benefit in limiting or slowing the community transmission of a pandemic virus by the use of combinations of partially effective NPIs. At the present time, this hypothesis remains unproven, and more work is needed before its validity can be established. (CDC 2007, p. 29).

## What is shaped?

The compartmental models discussed so far in this article are variants of the *SIR* model where the population is divided into three classes: *S*(*t*), the number of susceptibles, *I*(*t*) the number of infected, and *R*(*t*) the number of individuals who have been infected and then removed from the possibility of being infected again or of spreading infection. It is usually also assumed that the total of these three groups remains constant, that is *S* + *I* + *R* = *N*. The dynamics are usually described by the Eqs. (1)–(3). The solution of these equations was approximated by Kermack and McKendrick (1927) with a symmetric sech^2^ function. Today, computers can easily solve these equations numerically, the graphs of these solutions are still bell-shaped but not symmetrical. Nonetheless, the shape that is usually presented, like in all discussed figures of this article (Figs. 1, 2, 3, 4), is the symmetrical bell-shaped curve of the sech^2^ function. Kermack and McKendrick’s figure had become the emblematic curve of an epidemic.

The shape of Covid-19, however, differs from Kermack and McKendrick’s curve for the following reason. The downward movement of the *SIR* shape, that is the right side of the bell shape, is caused by an increase of the immune class *R*, that is the number of people who became immune of the disease. For Covid-19, this latter number is very low,^6^ thus cannot explain the downward movements of the graphs of Covid-19. In other words, the immune class *R* does not play a significant role in understanding the dynamics of Covid-19. A simpler model is needed, actually a *SIS* model, to describe a disease with no immunity against re-infection, in other words to indicate that the transit from individuals is from the susceptible class to the infective class and then back to the susceptible class.

Such a “simple model” that better describes the “essentials of the dynamical interaction” of Covid-19 is model A of Anderson and May (1981). This “simplest case” consists only of *S*(*t*), the number of susceptibles and *I*(*t*), the number of infected, so that *S*(*t*) + *I*(*t*) = *H*. The net rate of transmission of the infection is *βSI*, where *β* is the transmission parameter. Uninfected are assumed to die at the rate *b*. The parameter *α* represents the rate of disease-induced mortality, and *γ* is the assumed recovery rate. As a result, the rate of change in the number of infected individuals is

$$dI/dt = \beta SI{-} \, (\alpha + b + \gamma)I$$

If we rewrite this equation by taking into account that *S* = *H* − *I*, using the dimensionless variables *i* = *I*/*H* and *t’* = (*α* + *b* + *γ*)*t*, and the dimensionless reproduction rate $\mathscr{R}$ = *βH/*(*α* + *b* + *γ*), the dynamical equation becomes

$$di/dt^{^{\prime}} = i\left[ {\left( {R{-} \, 1} \right) \, {-}Ri} \right]$$

In representing the dynamics in the dimensionless variables and parameters, Anderson and May observed that: “It is immediately evident that the dynamics of the infection, the *shape* of the curve *i*(*t’*), depends only on the quantity $\mathscr{R}$. The scale of the time axis depends on (*α* + *b* + *γ*), and the absolute scale of the infected population *I* depends on *H*, but the qualitative nature of the host-parasite interaction here depends only on $\mathscr{R}$” (Anderson and May 1981, p. 460).

For the cases where $\mathscr{R}$ > 1 and is constant, the shape of the epidemic curve is that of a sigmoidal function, an upward sloping *S*-shaped curve that eventually approaches a steady value of *i* = 1 − 1/$\mathscr{R}$. For the cases where $\mathscr{R}$ = 1 or $\mathscr{R}$ < 1, and is constant, the shape is downward sloping, where *i*(*t*) decreases asymptotically to the value of 0. For $\mathscr{R}$ < 1 the slope is steeper than for $\mathscr{R}$ = 1. In other words, for all these cases, the slopes are proportional to ($\mathscr{R}$ − 1).

This means that when $\mathscr{R}$ is lowered, there is no bell-shape that is “flattened.” The shape of Covid-19 is not predetermined, it depends on the course of *R*. The curve moves up and down proportional to whether ($\mathscr{R}$ − 1) is positive or negative. Covid-19 also has no smooth course, which is suggested by the mathematical models, but its erratic shape is influenced by idiosyncratic social and political interactions. As the shape depends on how the measures for social distancing are followed, there is no ‘natural law’ that ensures that the curve will smoothly go down.

## Conclusions

The curve that needs to be flattened, the one published in *The Economist* of February 2020 and in the CDC *Guidance* is not an empirical curve but is the graphical representation of the mathematical solution of the differential equations of a *SIR* model. These equations describe the dynamics of an epidemic in the world of the model. It is a deterministic world and the closer this model is to reality, the better it can predict the development of a real epidemic. However to evaluate how close it is to reality or if it can be seen as a representation of an existing epidemic depends on a lot of empirical knowledge of the epidemic. This knowledge is not available when a new virus is identified to the world, such as the Corona virus.

Regarding the use of models, these warnings for policy design were made in the several planning guidances and discussions of mathematical models. For example, Mahmoud’s (2006) Letter Report contained a large section on the “strengths and weaknesses of the models presented, and strategies to improve predictive ability and usefulness”. Several of its recommendations pointed at the need for more empirical input. The usefulness of models is not only their representational role but equally important is their role to organise the relation between science and policy:

Models serve to organize and synthesize data from a variety of sources, identify data gaps, and to set priorities for further data acquisition. Modeling can also be used to promote dialogue between scientists, policymakers, and stakeholders about alternatives, uncertainties, assumptions and value judgments that underlie decisions. (Mahmoud 2006, pp. 3–4).

Anderson and May (1981, pp. 453–4) called their mathematical models “mathematical metaphors” that should be tested against empirical evidence. This means that with the appearance of a new epidemic one should try to find out which model is most appropriate to use. Nonetheless, the typical model includes the immune class *R*(*t*), which is responsible for the curve eventually going down (the right side of the bell shape). Maybe this is the reason why politicians believed that Covid-19 is a wave that will inevitably subside. The flattening was only meant to lower the pressure on hospitals.

To say that the only policy target is to lower the pressure on hospitals is not meant to undervalue its importance and relevance. The rationale of this target is to prevent overwhelming personnel and material resources of hospitals—which can increase morbidity and mortality—and to give additional time to develop effective treatment protocols, see the three goals in Fig. 3.^7^

There is also another important issue that makes that the slogan “flattening the curve” gives a wrong perception of the nature of control that plays a role with Covid-19. This has to do with the changing connotation of $\mathscr{R}_{0}$. It originated in demography, ecology and population genetics, and only recently moved to epidemiology. These natural science origins ensured that at the start $\mathscr{R}_{0}$ only captured the ‘natural’ characteristics of an epidemic, such as population densities. This had the implication that control of an epidemic only focused on changing these ‘natural’ characteristics. The natural world is usually assumed to be governed by natural lawlike relations. In the compartment models these ‘laws’ are represented by their dynamical equations. It is of no coincidence that the various models used to represent a purely biological epidemic match the data so well. Control in this context is indeed close to changing the parameters of the model, with a resulting change in the shape of the epidemic curve.

Since SARS $\mathscr{R}$ also came to capture social factors, it is important that epidemic models capture both natural relationships and social behaviour. This implies that control should also capture social behaviour which is not governed by natural laws. Also when social behaviour is controlled by *legal* laws, humans may not obey them or interpret them in different ways.

The curve of Covid-19 is not determined by natural laws which would imply that control is re-shaping the smooth curve of a phenomenon (“happening”) governed by these laws. The curve of Covid-19 is determined by the course of $\mathscr{R}_{0}$ which mainly reflects social and political attitudes. This not only makes the curve erratic but its future development unpredictable.

## Footnotes

1 Jones and Helmreich (2020) aptly articulate this combination as “an overlapping history of metaphorical, mathematical, and moral messaging.” They focus on the history of shaping epidemics as waves.

2 A comparable case of such a control is mid-twentieth century business cycle policy. The business cycle was presented as a smooth up-and-down going wave, and the aim was to lessen the wave’s amplitude (Boumans 2013).

3 The literature discussed in this article does not use consistently the same symbols for the compartments in question. To facilitate comparisons between the various discussed models, throughout this article the modern *SIR* notation is used. To distinguish this notation from the one used for the reproduction number, a script fond is used for the reproduction number (hence also for the “reproductive rate”) such that the reproduction rate appears as $\mathscr{R}$.

4 The sech function is a hyperbolic function (hyperbolic secant): ${\text{sech}}x = \frac{1}{{{\text{cosh}}x}} = \frac{2}{{e^{x} + e^{ - x} }}$. Hence, its curve has a symmetrical bell-shape.

5 I would like to thank Mick Roberts for bringing this point to my attention.

6 In July 2020 the percentage is not known yet, but it seems to be only a few percent.

7 I thank one of the anonymous referees for rightly emphasizing this point.
